# Minimally Invasive Simultaneous Colorectal and Liver Resection for Synchronous Colorectal Liver Metastasis—Short-Term Outcomes

**DOI:** 10.1007/s13193-024-01901-5

**Published:** 2024-02-23

**Authors:** Kunal Nandy, Mufaddal Kazi, Shraddha Patkar, Gurudutt Varty, Ashwin De Souza, Avanish Saklani, Mahesh Goel

**Affiliations:** 1 Division of Hepatobiliary Surgical Oncology, Department of Surgical Oncology, Tata Memorial Hospital, Homi Bhabha National Institute, Parel, Mumbai, Maharashtra India; 2 Department of Surgical Oncology, Tata Memorial Hospital, Homi Bhabha National Institute, Parel, Mumbai, Maharashtra India

**Keywords:** Laparoscopy, Minimal access, Hepatectomy, Colorectum, Synchronous

## Abstract

Surgical management of colorectal disease and liver metastatectomy can be staged or synchronous. A minimally invasive approach in synchronous resection in the selected group of patients may improve postoperative outcomes. The present study aimed to explore the safety and feasibility of simultaneous liver and colorectal resection for synchronous metastasis by a minimally invasive approach in terms of major morbidity and R0 resection rates. The present study is a retrospective review of a prospectively maintained database. All patients who underwent minimally invasive simultaneous resection of colorectal malignancy and liver metastases between January 2020 and April 2023 were included. A total of 39 patients were included in the study. The median age was 54 (23–79) years with 28 male (72%) and 11 female (28%) patients. Rectum (*n* = 21, 54%) was the most common primary location. The most commonly performed procedures were low anterior resection (*n* = 12) and parenchymal sparing non-anatomical resection (*n* = 23, 59%). The median surgery duration was 280 (150–520) min, and the median blood loss was 400 (50–2100) ml. The median hospital stay was 7 (5–18) days. Five (12.6%) patients had major complications. With a median follow-up of 12 months, the 2-year overall survival (OS) and disease-free survival (DFS) were 84.6% and 37%, respectively. Simultaneous liver and colorectal resection by minimal access approach is feasible in selected groups of patients depending on the extent of hepatectomy, the patient’s general condition, and surgical team experience. A minimal access approach leads to faster recovery without compromising on the oncological radicality.

## Introduction

Colorectal cancer (CRC) is among the second most common cancers in women and the third most common in men across the world and represents a major healthcare burden [1]. The liver is the most common site of metastasis and up to 70% of patients with CRC develop liver metastasis during their disease course [2]. Around 18–25% of patients have synchronous hepatic metastasis detected at the time of initial presentation [3]. The approach to managing synchronously detected colorectal liver metastases (CRLM) has been debated for a long time with no concrete consensus on sequencing and timing liver resection and colorectal surgery. Both simultaneous resection and staged resection approaches have been studied with no oncological advantage of either approach over others [4]. The strategy of “one-step procedure” where hepatic and colorectal resection is done in the same setting has demonstrated significant advantages in terms of lower complication rates, shorter hospital stays, and a decrease in cost [5]. The major problem with simultaneous resection is its associated morbidity of surgery especially when major hepatectomy is involved [6].

In an earlier study, we found an unacceptable level of morbidity (≥ 40%) when simultaneous major hepatectomy was combined with colorectal resections in patients with medical comorbid conditions [6]. Since then, major hepatectomies have been carefully considered for simultaneous resections, when the predicted probability of major complications was limited to 20%. The other change has been the incorporation of minimally invasive approaches for synchronous metastasis with greater frequency with the ultimate aim of reducing morbidity.

In the past decade, there has been increasing experience in performing hepatectomy by minimal access approach demonstrating safety and feasibility [7–9]. This has pushed surgeons to offer a single-step minimally invasive approach to managing colorectal surgery and liver resection. The multi-societal European consensus statement for synchronous CRLM considers minimally invasive surgery (MIS) as an acceptable option. However, the majority of the literature has focused on MIS hepatectomy, rather than MIS simultaneous resections [10].

The present study aimed to explore the safety and feasibility of colorectal resection and simultaneous minor hepatectomies of varying difficulties for synchronous metastasis by a minimally invasive approach in terms of major morbidity and R0 resection rates.

## Materials and Methods

The present study is a retrospective review of a prospectively maintained database. All patients who underwent simultaneous resection of colorectal malignancy and liver metastases via a minimally invasive approach between January 2020 and April 2023 were included. All patients underwent colonoscopy, biopsy, and contrast-enhanced computed tomography (CECT) of the chest, abdomen, and pelvis. Magnetic resonance imaging (MRI) was done for all patients with rectal cancers. Positron emission tomography (PET) was performed selectively to rule out extra-hepatic diseases.

Liver lesions were evaluated using a combination of triphasic CECT, MRI, and sonography based on the discretion of the multidisciplinary team (MDT). All decisions regarding simultaneous resections were taken in MDT discussions involving surgical oncologists, radiation oncologists, medical oncologists, and interventional radiologists. The decision for perioperative chemotherapy was individualized for resectable liver metastasis based on the need for preoperative therapy in advanced rectal cancers and the number, size, and distribution of liver metastasis. Fong’s clinical score and KRAS/BRAF mutation status were taken into consideration while deciding on preoperative chemotherapy [11].

MIS included laparoscopic and robotic approaches on the Da Vinci Xi platform. Importantly, operations were performed without changing the patient position for the liver and colorectal procedures, irrespective of the site of metastasis in the liver or the primary tumor. The technique for single-position multi-quadrant surgery has been previously described by our group, including for those in the posterior-superior segments [12–14]. As part of a simultaneous operation, the liver resection was always carried out first, with or without portal clamping, followed by the colorectal resection.

Non-anatomical resections or parenchymal preserving surgery was performed whenever feasible. The IWATE scoring system was used to grade the difficulty levels of laparoscopic liver resection [15]. Resectability of liver metastasis was defined as the ability to achieve R0 resection with an adequate FLR. Post-surgery liver-directed therapy in the form of ablation or hepatic artery infusional chemotherapy (HAIC) was administered after a multidisciplinary discussion. Small (< 3 cm) deep-seated lesions whose resection would sacrifice large volumes of functioning liver were not subjected to surgical excision and would instead be ablated after resecting other liver metastases. HAIC is delivered as an adjuvant for patients at high risk for liver recurrences as part of an ongoing study.

### Outcome

Perioperative outcomes recorded were blood loss, hospital stay, 30-day postoperative complications, and 90-day hospital readmissions. Complications were recorded as per the Clavien–Dindo scale [16]. Grade IIIA or higher complications were regarded as major morbidity. Liver-specific complications like post-hepatectomy bile leaks, liver failure, and hemorrhage were recorded as per the International Study Group of Liver Surgery (ISGLS) classification system [17–19]. Colorectal anastomotic leak was defined clinically or based on radiological extraluminal contrast extravasation. The safety threshold was defined at 20% major morbidity rate based on our previous experience as mentioned before [6]. Thus, simultaneous resection would be considered unsafe if significant complications were more than 20%. R0 resection for colorectal resections was considered for a tumor-free margin of > 1 mm while that for liver resections was considered when the tumor was absent at the inked resection surface. Overall survival (OS) was calculated from the date of operation to the date of death or last follow up whichever occurred first. Disease-free survival (DFS) was calculated from the date of operation to the date of recurrence or death.

### Statistics

The descriptive analysis included median and interquartile ranges in continuous variables, while categorical and ordinal variables were reported as counts with proportions. Time-to-event variables were described using the Kaplan–Meier curve, and the median follow-up was recorded using the inverse Kaplan–Meier method. All statistical analyses were performed using SPSS Statistics version 24.0 (IBM, Armonk, NY, USA).

### Ethics

The data of the present study were collected in the course of common clinical practice, and accordingly, the signed informed consent was obtained from each patient for any treatment or clinical procedure. The study protocol was in accordance with the ethical standards of the institutional research committee.

## Results

During the study period, 39 patients were included in the study. Median age was 54 (23–79) years, and 28 (72%) were males. Median body mass index (BMI) was 22.8 (14.33–32.45) kg/m^2^. Rectum (*n* = 21, 54%) was the most common primary location followed by the left colon (*n* = 12, 31%). The median size of liver metastasis was 2.8 (2–3.7) cm, and the median number of metastases was 2 (1–8). Twenty-seven (69.2%) patients received neoadjuvant treatment (Table 1).

**Table 1:** Baseline characteristics

Characteristic	*N* = 39
Age (years); median (range)	54 (23, 79)
Sex	
Male	28 (72%)
Female	11 (28%)
Body mass index (kg/m^2^); median (range)	22.8 (14.33, 32.45)
Carcinoembryonic antigen (ng/ml); median (range)	12 (1, 899)
Comorbidities (American Society of Anaesthesiologists)	
1	18 (46%)
2	19 (49%)
3	2 (5.1%)
Colorectal cT stage	
T2	3 (7.7%)
T3	21 (54%)
T4	15 (38%)
Colorectal N stage	
N0	1 (2.6%)
N1	22 (56%)
N2	16 (41%)
Other M1 sites	
None	31 (79%)
Lung	4 (10%)
Nodal	2 (5.1%)
Peritoneum	1 (2.6%)
Multiple	1 (2.6%)
Location of the primary tumor	
Right colon	6 (15%)
Left colon	12 (31%)
Rectum	21 (54%)
Histologic differentiation	
Well-/moderately differentiated	37 (95%)
Poorly differentiated	2 (5.1%)
Signet-ring cell cancer	1 (2.6%)
Number of liver metastasis; median (range)	2 (1, 8)
Size of largest liver metastasis (cm); median (range)	2.80 (2.00, 3.70)
Fong’s clinical risk score	
1	1 (2.6%)
2	13 (33%)
3	20 (51%)
4	4 (10%)
5	1 (2.6%)
Neoadjuvant therapy	
None	12 (31%)
Chemotherapy	9 (23%)
Radiation	3 (7.7%)
Chemotherapy and radiation	15 (38%)
Chemotherapy cycles (*n* = 24); median (range)	4 (4, 20)
Chemotherapy regime (*n* = 24)	
FOLFOX/CapeOx	17 (71%)
FOLFIRINOX	7 (29%)
Biologicals	
None	33 (85%)
Panitumumab	1 (2.6%)
Bevacizumab	5 (13%)
RAS (*n* = 17)	
Wild	12 (71%)
Mutant	5 (29%)
RAF (*n* = 17)	
Wild	15 (88%)
Mutant	2 (12%)
MSI (*n* = 23)	
Stable	22 (96%)
High	1 (4.3%)

*RAS* rat sarcoma virus, *RAF* rapidly accelerated fibrosarcoma, *MSI* microsatellite instability, *FOLFOX-5* fluorouracil + oxaliplatin, *FOLFIRINOX-5* fluorouracil + oxaliplatin + irinotecan, *CAPEOX* capecitabine + oxaliplatin.

Low anterior resection (*n* = 12) and left colectomy/anterior resection(*n* = 12) were the most commonly performed colorectal surgery, and the most frequent liver resection performed was parenchymal sparing non-anatomical resection (*n* = 23, 59%) followed by anatomical mono-segmentectomy (*n* = 8,21%), left lateral hepatectomy (*n* = 6, 15%), and posterior sectionectomy (*n* = 2, 5.1%). Laparoscopic surgery was performed in 32 (82%) patients, and 7 (18%) patients underwent surgery via a robotic approach. Apart from colorectal and liver resection, lateral pelvic node dissection (*n* = 4), cytoreductive surgery (*n* = 2), and retroperitoneal lymph node dissection (*n* = 1) were other adjunct procedures performed. The median IWATE difficulty score was 5 (1–10) with the majority in intermediate level (*n* = 16, 41%) followed by low (*n* = 14, 36%). There were no conversions to open operations (Table 2).

**Table 2:** Operative features

Characteristic	*N* = 39
Colorectal surgery	
Right colectomy	6 (15%)
Left colectomy/high-anterior resection	12 (31%)
Low anterior resection	12 (30.8%)
Intersphincteric resection	2 (5.1%)
Abdominoperineal resection	5 (12.8%)
Total pelvic exenteration	2 (5.1%)
Ostomy (permanent/temporary)	23 (59%)
Adjunct operations	
Cytoreduction ± intraperitoneal chemotherapy	2
Retroperitoneal lymph node dissection	1
Lateral pelvic node dissection	4
Liver surgery	
Anatomic resection	8 (21%)
Non-anatomic resection	25 (64%)
Both anatomic and non-anatomic resections	6 (15%)
Surgical approach	
Laparoscopic	32 (82%)
Robotic	7 (18%)
Pringle duration (min); median (range)	20 (0, 65)
Number of segments removed	
1	21 (54%)
2	13 (33%)
3	2 (5.1%)
4	3 (7.7%)
Liver operation descriptions	
Left lateral sectionectomy	6 (15%)
Parenchymal sparing non-anatomic resection	23 (59%)
Anatomic mono-segmentectomy	8 (21%)
Posterior sectionectomy	2 (5.1%)
IWATE difficulty score components	
Segment score	
S3	2 (5.1%)
S2/S6	12 (31%)
S4/S5	10 (26%)
S7/S8	15 (38%)
Extent score	
Partial resection (Hr0)	23 (59%)
Hr-LLR (left lateral sectionectomy)	6 (15%)
Segmentectomy (Hr-S)	8 (21%)
Not less than a sectionectomy (Hr-1, 2)	2 (5.1%)
Size score	
< 3 cm	28 (72%)
≥ 3 cm	11 (28%)
Proximity score	5 (13%)
Liver function score—cirrhosis	0
IWATE difficulty score; median (range)	5 (1, 10)
Difficulty level	
Low (1–3)	14 (36%)
Intermediate (4–6)	16 (41%)
High (7–10)	9 (23%)
Complications	
Operative time (mins); median (range)	280 (150, 520)
Blood loss (ml); median (range)	400 (50, 2100)
Duration for liver surgery (mins); median (range)	110 (15, 240)
Blood loss during liver resection (ml); median (range)	100 (10, 2000)
Duration of colorectal resection (mins); median (range)	150 (50, 480)
Blood loss during colorectal resection (ml); median (range)	150 (10, 1450)
Hospital stay (days); median (range)	7 (5, 18)
Clavien–Dindo postoperative complication grade	
0	25 (64%)
I	5 (13%)
II	4 (10%)
IIIb	4 (10%)
IV	1 (2.6%)
Readmission within 90 days	2 (5.1%)
Anastomotic leaks (*n* = 34)	3 (8.8%)
Bile leak	0

The median surgery duration was 280 (150–520) min with a median blood loss of 400 (50–2100) ml. The median hospital stay was 7 (5–18) days. Five (12.6%) patients had major complications with a Clavien–Dindo of more than III. Two (5.1%) patients had re-admission within 90 days. Three (8.8%) patients had anastomosis-related complications, and there was no complication related to liver resection (Table 2). One (2.6%) and 4 (10.3%) patients had microscopic margin positive resection in the colorectum and liver resections, respectively.

Thirty-six (92%) patients received adjuvant systemic therapy. Post-surgery liver-directed therapy was done in 14 (36.2%) patients with the most common procedure being ablation (*n* = 10, 26%), TACE (*n* = 2, 5.1%), and HAIC (*n* = 2, 5.1%) (Table 3). At a median follow-up of 12 months, 16 (41%) developed recurrence of which 1(2.6%) had a recurrence in the local colorectal region and 7 (18%) had in the remnant liver. Two-year overall survival (OS) and disease-free survival (DFS) were 84.6% and 37%, respectively.

**Table 3:** Pathological and oncological outcomes

Characteristic	*N* = 39
pT	
0	1 (2.6%)
2	5 (13%)
3	23 (59%)
4	10 (26%)
pN	
0	13 (33%)
1	11 (28%)
2	15 (38%)
R status colorectal	
R0	38 (97%)
R1	1 (2.6%)
R status liver	
R0	35 (89.7%)
R1	4 (10.3%)
Adjuvant systemic therapy	36 (92%)
Adjunctive liver therapy	
None	25 (64%)
Thermal ablation	10 (26%)
Trans-arterial chemo-embolization	2 (5.1%)
Hepatic artery infusional chemotherapy	2 (5.1%)
Median follow-up	12 months
Recurrences	16 (41%)
Local recurrence	1 (2.6%)
Liver recurrence	7 (18%)
Death	4 (10%)
2-year disease-free survival	37%
2-year overall survival	84.6%

## Discussion

Management of colorectal liver metastasis is multimodal involving surgery, radiation, and systemic therapy. When surgical management is planned for synchronous CRLM the timing and sequencing with chemotherapy and radiation becomes challenging and important. The METASYNC randomized controlled trial looked at the comparison between synchronous and delayed liver metastasectomy and found that significant morbidity was similar in both groups and survival tended to be better in the synchronous resection arm [20]. Synchronous resections have reduced hospital stays since both procedures are done in the same admission, hence reducing cost also. Critics of simultaneous resection argue that combining clean surgical procedures like hepatectomy with colorectal resection increases the risk of contamination and thus can result in higher morbidity rates [21]. Another concern was related to intestinal edema after portal clamping and vulnerability to infection due to post-operative impaired liver function which could have some effect on colorectal anastomosis [22, 23]. The presence of comorbidities and the requirement of major hepatectomies were associated with increased levels of post-surgery morbidity [5, 6]. Major hepatectomy is not a contraindication to simultaneous resection but a careful patient selection is recommended.

Laparoscopic colorectal resections for carcinoma have been well-standardized and are currently among the most widely utilized approaches for colorectal resections [23]. Hence, it becomes important to expand the utility of the minimal access approach in liver resections. Advantages of the laparoscopic approach include a better postoperative course leading to early discharge and faster return to normal activities. There are reduced incidences of hernia due to less trauma to the abdominal wall. There is still continuing debate over the indications and drawbacks of combined colorectal and liver resections by minimal access approach.

In the present series of 39 minimally invasive simultaneous resections of colorectal cancer and liver metastasis, the safety threshold was not crossed with a major complication rate of 12.6%. Berti et al. [5] reported their experience of total laparoscopic synchronous resection in 35 patients with the majority of liver resections being minor parenchymal sparing wedge resections. Significant morbidity in their series was 20% compared to 12.6% in the present series. In a multicentre international series of 142 simultaneous resections by Ferretti et al. [24], with a conversion rate of 4.9%, the significant morbidity rate was 31% with a mortality of 2.1%. The majority of the patients underwent minor hepatectomies (88%). Post-hepatectomy complications like bile leak, liver failure, ascites, and hemorrhage were encountered in 7.7% of patients. Although the study is non-comparative, the median blood loss and hospital stay were better in the present series compared to historical controls [5, 24]

Based on our prior experience that showed high morbidity combining major hepatectomy and colorectal surgery, we included only minor liver resections operated synchronously in the present study [6]. Hence, to begin with, we restricted to liver resections of multiple non-contiguous segments of at most two contiguous segments; with increasing experience and controlled morbidity, we plan to expand the services to major liver resections as well.

In the present study, a validated IWATE score was used. The majority of the patients were in the intermediate level (*n* = 16, 41%) followed by low (*n* = 14, 36%). Tumor location was never a contraindication for offering a minimal access approach. Thus, posterior-superior and those in difficult segments were also operated upon if they could be ease easily localized or encompassed in an anatomic resection. The results achieved are also due to careful patient selection keeping in mind the learning curve and technological restrictions.

Colorectal resections spanned the entire spectrum from standard colectomies to rectal resections and also included exenteration operations. The major complications (8.8%) encountered were all related to the colorectal resection rather than the liver resection which was well-controlled.

Limitations of the study include the retrospective nature with a small sample size. However, this is among the largest single-center simultaneous series and the first from the Indian subcontinent. In the present series, there was selection bias in terms of selecting relatively less complex liver resections to limit morbidity. Follow-up in the current series was also small to understand the effect of minimal access surgery on oncological outcomes. Finally, this is not a comparative study with the conventional open approach.

## Conclusion

Simultaneous liver and colorectal resection by minimal access approach is feasible in the selected group of patients depending on the location of the tumor, the extent of resection, and the experience of the surgical team. A minimal access approach does not increase post-operative morbidity and leads to faster recovery without compromising on the oncological principles of surgery.

## Data Availability

The datasets generated and/or analyzed during the current study are available from the corresponding author upon reasonable request.
